# Non-Invasive Prenatal Testing beyond Trisomies

**DOI:** 10.25122/jml-2019-0053

**Published:** 2019

**Authors:** Ioan Dumitru Suciu, Oana Daniela Toader, Slavyana Galeva, Lucian Pop

**Affiliations:** 1. Department of General Surgery, Floreasca Emergency Hospital, Bucharest, Romania; 2. Carol Davila University of Medicine and Pharmacy, Bucharest, Romania; 3. Department of Obstetrics and Gynecology, Alessandrescu-Rusescu Institute of Mother and Child Care, Bucharest, Romania; 4. Department of Obstetrics and Gynecology, Il Sagbal Sheynovo Hospital, Sofia, Brunei Darussalam

**Keywords:** NIPT, Amniocentesis, genome, chromosomal abnormalities

## Abstract

The last decade has seen incredible advances in the genetic era, in next-generation sequencing of cell-free DNA in the maternal plasma, detecting abnormal fetal chromosomes. Non-invasive prenatal testing (NIPT) has showed increased sensitivity and specificity for Down syndrome superior to any other screening test. Technical advances have made possible the detection of other conditions which does not necessarily mean clinical benefit for the patient. Private laboratories have added multiple conditions in the panel of NIPT, but some of these abnormalities are so rare, that their prevalence is not even clear. Data regarding clinical performance of extended NIPT is lacking. Implementation of such a test has to be carefully weighed, and not only the benefits but also the harm should be taken into account.

## Introduction

Screening for Down syndrome started in the ’70s relying on the observation that Down syndrome is associated with advanced maternal age. Screening age was set up at 40 years old, but later, when the risk of amniocentesis was well-ascertained, the screening age was lowered at 35 and older, encompassing 5% of all women with a 30% detection rate of babies with Down syndrome [1]. Now, more than 40 years after its introduction, screening for trisomy 21 has entered into the era of non-invasive prenatal testing.

Discovered in 1997 by Denis Lo, introduced into clinical practice in 2011, mainly by private companies, using circulating free DNA (cfDNA) for NIPT (non-invasive prenatal testing) has become a reality in routine antenatal care and its features are extending on regular basis [2]. Even before screening for trisomies using NIPT was implemented, some laboratories were offering the detection of Y chromosome sequence in maternal blood through quantitative polymerase chain reaction in families with conditions related to the Y chromosome [3]. Recently, the availability of genome-wide array extended the application of NIPT for rare trisomies and CNVs (copy number variants). Before implementation, advantages and drawbacks of screening must be carefully assessed when considering the introduction of NIPT in the general population. The World Health Organization (WHO) has established the acceptance criteria and conditions for screening a long time ago, being recently updated (Table 1) [4]. However, NIPT hardly fulfills these criteria beyond trisomies [5].

**Table 1:** WHO criteria for NIPT screening

The condition should be an important health problem.
The natural history of the condition should be adequately understood
Facilities for diagnosis and treatment should be available
The overall benefits of screening should outweigh the harm.
There should be scientific evidence regarding screening program effectiveness.
The screening program should respond to a recognized need.
The objectives of screening should be defined at the outset.

### NIPT for Trisomies

In the last five years, numerous studies have focused on the clinical applicability of NIPT in common trisomies and sex chromosome aneuploidies. Although the detection rate (DR) for trisomy (T) 21 is extremely high (over 95%) in all studies, its accuracy regarding the DR for T18 and T13 has come under scrutiny, particularly when looking at subgroups and not high-risk population. In the meta-analysis published by Taylor-Phillips at the request of the National Institute of Clinical Excellence, the DR for Down syndrome was 96%, 87% for T18 and 77% for T13 when looking at the general population [6]. The Netherlands and Belgium have introduced NIPT as a first-tier in screening, and preliminary data suggest a lower accuracy for Patau syndrome (52%) [7]. Nevertheless, NIPT is currently expanding as the price is going down, and more conditions are being added to the panel.

### Sex chromosome abnormalities (SCAs)

These are common conditions. Prevalence of sex chromosomes abnormalities is around 1.88/10000 births. Ruling out Turner syndrome, these conditions do not present any symptoms in the immediate period following delivery. Testing for monosomy X has been available since 2012 and was initially offered to women showing ultrasound features of Turner syndrome. This was followed by the introduction of other conditions linked to the sex chromosomes allowing the detection of XXX, XXY, and XYY [3]. The DR for monosomy X is 89% and 82% to 90% for Diplo Y, Klinefelter and Triple X syndromes [8]. SCAs have a particular behaviour with a predisposition to cell line mosaicism, which has a deep impact on the subsequent management and diagnosis. One of the largest studies regarding SCA was published by Grati et al. in 2017[9]. This study took into account the results of 522 patients. Confined placental mosaicism occurred in 23.4% of cases without ultrasound anomalies giving a positive predictive value (PPV) of 53% in these cases. PPV was 98.9% in cases with ultrasound anomalies. Whenever there is a positive NIPT for SCAs without ultrasound features, testing through amniocentesis is recommended and this should be bear in mind when counseling future parents [9]. Although testing for SCAs is fairly accurate, this test does pose an ethical dilemma in terms of sex selections, the reason for which the Netherlands (one of the countries that have introduced NIPT as a first-tier) has excluded SCAs from testing, despite its usefulness in conditions like Duchenne muscular dystrophy or the adrenogenital syndrome. Also, professional societies like the European and American Societies of Human Genetics currently recommend against the use of NIPT for SCAs [7, 10, 11].

### Rare trisomies and microdeletions/duplications

With a prevalence of 0.3-0.8%, rare autosomal trisomies (RAT) represent either placental mosaicism or uniparental disomy, cases in which the fetuses inherit both chromosomes from one parent [12]. In a study published by Pertile et al., only 5 cases out of 60 were true positive RATs, the others being cases of confined placental mosaicism[13]. Excepting confined placental mosaicism (CPM) for trisomy 16, where the risk for the fetus is well established, there is no data regarding other chromosomes involved in CPM [14]. Pathological CNVs occur in 1.7% of pregnancies with normal findings and are more common at a younger age than trisomies [15]. CfDNA companies have extended the panel of conditions and included Di George, Cri du Chat, Prader Willi/Angelman syndromes, 1p36 deletion, Jacobson syndrome, Wolf Hirschhorn syndrome. Di George syndrome represents the second cause of mental retardation in children, after Down syndrome. There is a little rational basis for screening other conditions which are exceedingly rare [14]. Proper counseling is of utmost importance in fetal medicine. Future parents must be able to understand and to process the information provided. One of the critical questions for parents is why did this happen, and since the natural history for many of these conditions is unknown, it is challenging to answer. It might present with clinically significant anomalies, or it might have an insignificant effect[16]. There are over 2100 CNVs, majority of them extremely rare and even genome-wide array of cfDNA can’t detect the majority of them due to their small size. At the moment, cfDNA testing can detect imbalances of at least 3Mb up to 6 Mb [17]. Reliable detection rates cannot be calculated based on the available data. The majority of articles are based on retrospective cases, where samples collected before deliveries were tested in cases of newborns with rare aneuploidies [18].

### Triploidies by NIPT

Triploidies are characterised usually by a very thin placenta and subsequently by a very low cfDNA, therefore making triploidy extremely difficult to be detected through NIPT, even in the presence of scan features and abnormal biomarkers which are suggestive for triploidy in up to 90% of cases [19].

### Fetal Blood Group

Amniocentesis has become outdated as a diagnosis method for foetal blood group, with NIPT having an accuracy of 99.7% according to Chitty et al. Additionally, NIPT is useful not only for detecting the rhesus status but also for predicting the fetal red cell antigen status for, C, c, E, e and Kell (K).

### Monogenic disease

In terms of monogenic conditions, achondroplasia was the first monogenic disease that was detected thorugh NIPT and introduced into clinical practice. Ever since, the spectrum of mongenic disease has extended including conditon like Cruzon syndrome, thanatophoric dysplasia, osteogenesis imperfecta, Apert syndrome, cystic fibrosis, torsion dystonia, Fraser syndrome and many others [15]. Clasically, with the advancement of next-generation sequencing, it is possible to describe cell-free fetal DNA in maternal plasma. The detection is not straightforward for monogenic conditions, particularly in the case of maternally-inherited alleles due to the fact that that this inherited allele is genetically identical with one of the maternal allelels from the cfDNA and this hampers direct observation of maternally-inherited alleles. Although NIPT detection of paternally-inherited monogenic conditions is already in use, the same cannot be said for maternally-inherited conditions altough research studies are promising by using targeted haplotyping [21].

### NIPT fetal fraction

Approximately 2-3% of all NIPTs have an inconclusive result [22]. There is a number of causes for this, such as collection, transportation and storage, technical failure, maternal and fetal mosaicism, as well as maternal malignancy. There are laboratories that do not measure the fetal fraction, but previous studies illustrate that a fetal fraction below 3-4% increases the chances of getting no report. By not calculating the fetal fraction, those laboratories are increasing the chances of giving a false result as it was demonstrated by Takuda and colleagues who showed that one laboratory provided a normal fetal result for a non-pregnant woman [23]. On average, the fetal fraction is around 10% and differs among individuals, being directly proportional to gestational age and inversely proportional to maternal weight [24]. In patients with small placentas, the risk of getting no result is increased, which is obvious in cases of trisomy 13 and trisomy 18, PAPP-A and beta-hCG being also extremely low in these cases. The fetal fraction is dependent on ethnicity as well as it is lower in women of Afro-Caribbean origin compared to Caucasians, lower in IVF pregnancies and increases with fetal crown-rump length, serum pregnancy-associated plasma protein-A, serum-free β-human chorionic gonadotropin, smoking and trisomy 21 karyotype, as it was shown by Ashoor and colleagues [25]. Receiving a no result report increases the chances of a negative outcome such as miscarriage, abnormal chromosomes, preeclampsia, and gestational diabetes mellitus [26].

### Screening in multiple gestations

Although there is a growing number of studies regarding singletons, there is a limited number of articles addressing the performance of NIPT in twins. The role of cfDNA as a biochemical marker in a combined screening test has one shortcoming in twins, the fact that the euploid fetus might mask the abnormal one. However, the results of these studies are encouraging, reporting a detection rate similar to the one in singletons and higher than the combined screening test [27].

### Counseling in NIPT

Genetic counseling is not easy. In a prenatal setting, it requires efficient communication, details and support among all parties. Advances in genetics have made prenatal counseling even more complex and time-consuming. Future parents should also have the opportunity to deliberate following counseling.

It is truly remarkable how cfDNA has been accepted and promoted by women, taking into account its commercial nature and a rather high price for the amount of information which is being provided.

In the dawn of cfDNA as a prenatal test, there were concerns regarding its use as a possible diagnostic test. This issue was sorted out by recommending NIPT as a screening test and not as a diagnostic test. Patients should be provided with written information, and in addition to this, the healthcare provider should discuss the details and emphasize the limitations of this test, its sensitivity, false-positive rate, negative and positive predictive value, incidental findings, timing and subsequent management. Unquestionably, antenatal counseling is time-consuming, and this will add extra duties for the healthcare professionals, which is essential for a meaningful decision [28].

## Conclusions

The introduction of NIPT has changed screening and antenatal care in the genomic era. Advances in prenatal screening are driven by technological progress and not by a specific condition. This fact has also brought a lot of ambiguity because of the lack of robust data in terms of NIPT beyond trisomies. The majority of non-recurrent CNVs or small-size CNVs cannot be picked up by NIPT, giving an artificial sense of security. Screening for multiple conditions is increasing the false positive rate which will also increase the number of invasive procedures, canceling the main advantage of NIPT, which is a false positive rate of 0.2% compared to 4% compared to the combined screening test [14, 29]. Many patients have never heard about micro-deletions, sex chromosomes abnormalities, and most doctors do not spend time explaining their significance to the patient, creating more confusion than clarity for the parents. Expanding NIPT for more conditions means a higher cost, which implies unequal access to screening. The significance of a test translates in its clinical utility and its capacity to ease decision-making and NIPT beyond trisomies does not fulfill this requirement. Whole-genome NIPT is the right idea, but more progress needs to be made.

## Conflict of Interest

The authors confirm that there are no conflicts of interest.
